# Content analysis of state-level review materials for K-2 core literacy curricula

**DOI:** 10.1007/s11881-025-00334-1

**Published:** 2025-06-05

**Authors:** Alisha Demchak, Katlynn Dahl-Leonard, Emily J. Solari, Colby Hall, Stephanie Tatel, Katie E. Wilburn

**Affiliations:** 1 Department of Curriculum, Instruction, and Special Education, University of Virginia, Charlottesville, USA; 2 Department of Curriculum and Instruction, University of Houston, Houston, USA

**Keywords:** Content analysis, Curriculum review, Literacy curricula, Literacy legislation

## Abstract

Within the last two decades, several states have enacted literacy-related legislation with the goal of improving national literacy outcomes for students. In alignment with this legislation, some states have undertaken a core literacy curriculum review process. These processes often involve the development and implementation of an evaluation system (e.g., rubric, checklist), resulting in a list of approved, high-quality, core literacy curricula that school systems are either required or recommended to adopt. However, little is known about the materials states use to review core literacy curricula. This study sought to identify which states engage in the review of K-2 core literacy curricula and to examine the materials they use to identify high-quality curricula. A content analysis of K-2 core literacy curriculum review materials from 24 states revealed large variability across materials. Overall, there was preliminary support of evidence-based content and pedagogy reflected in the materials. At the same time, there was potential for better alignment with some evidence-based practices. Further examination of K-2 core literacy curriculum review materials and the processes in which they are used is crucial to ensure clarity and consistency for educators, policymakers, and publishers. With sustained focus on evidence-based approaches and cross-state collaboration and alignment, there exists the potential to create meaningful and lasting improvements in literacy instruction and outcomes for all students.

**Supplementary Information:**

The online version contains supplementary material available at 10.1007/s11881-025-00334-1.

Reading and writing proficiency is closely linked to academic achievement, vocational opportunities, and physical and mental health (Kutner et al., 2007). Conversely, underdeveloped literacy skills can have profound negative consequences. For example, compared to their peers, students with reading and writing difficulties are more likely to experience truancy and expulsion, and drop out of high school (Kutner et al., 2007). They also have an increased likelihood of adverse outcomes later in life, including incarceration and poverty (Ritchie et al., 2013). Recognizing the critical role of literacy in a wide range of individual and societal outcomes, researchers from education, psychology, and neuroscience have worked to uncover how literacy develops and identify instructional practices that benefit students. Despite these efforts, national reading and writing scores in the United States (U.S.) remain persistently low (National Assessment of Education Progress, 2024; National Center for Education Statistics, 2012), and significant disparities persist between students from marginalized communities and their peers (de Brey et al., 2019).

National and state legislatures have invested significant resources into efforts to address the problem of persistent low literacy achievement. For example, beginning in the 1990s, federal initiatives, such as *No Child Left Behind* and *Reading First*, aimed at influencing literacy instruction and improving student outcomes. Later, the *Every Student Succeeds Act* (ESSA) built on these efforts by aiming to grant states more flexibility while maintaining a focus on ensuring better outcomes for all students*.* These initiatives prompted many states to enact literacy legislation that has resulted in multifaceted state-level policies targeting pre-service teacher preparation, professional development for in-service teachers, hiring and training of specialized personnel (e.g., reading specialists), and instruction and assessment practices (Holston, 2024; Neuman et al., 2023; Peak, 2022). Since 2013, more than 40 states have implemented policies related to literacy instruction, including some that focus on curriculum adoption (Schwartz, 2022). States’ curriculum adoption policies often include a curriculum review process in which an evaluation system (e.g., rubric, checklist) is developed and implemented, resulting in a list of approved literacy curricula that school systems are either mandated or encouraged to adopt. Although research suggests that the use of high-quality (i.e., research evidence-based) core literacy curriculum ensures equitable instruction and positively impacts student learning (Chingos & Whitehurst, 2012; Clements, 2007), little is known about the materials (i.e., evaluation systems, such as rubrics and checklists, as well as accompanying process documents) states use to review core literacy curricula.

## Curriculum as a reform tool

A curriculum, broadly defined as organized educational experiences, materials, and guidance provided to students, can be a powerful lever in shaping instruction and student learning outcomes, and thus an important tool during efforts to reform educational systems. A curriculum defines not only *what* to teach but also *how* to teach it. Adherence to high-quality curricula can thus ensure that students engage with content that research determines is important for students to learn and with evidence-based instructional methods (Steiner et al., 2019). Further, by organizing learning objectives and sequencing topics logically, a curriculum can provide students with access to standards-aligned instruction that is horizontally and vertically coherent, providing continuity in learning as students’ progress through grade levels (Folsom et al., 2017; Polikoff, 2012). Finally, in addition to directly influencing student outcomes, high-quality curricula can have a meaningful indirect impact on student learning by increasing teacher knowledge (i.e., their understanding of content and pedagogy; e.g., Davis & Krajcik, 2005; Valencia et al., 2006).

Although there are a wide variety of core literacy curricula in use in the U.S. today (Schwartz, 2022), variability in alignment between core literacy curricula and literacy research has long been documented by researchers, with many programs lacking adequate integration of evidence-based practices (Reutzel et al., 2014; Seidenberg et al., 2020). For example, Reutzel and colleagues (2014) conducted a content analysis of the five top-selling core reading curricula used in the U.S. They examined the reading programs for elements of explicit instruction (i.e., direct explanation, modeling, guided practice, discussion, feedback, independent practice, and monitoring) in essential areas of reading instruction (i.e., fluency, vocabulary, comprehension, phonics, and phonemic awareness; National Reading Panel, 2000). Their study revealed that although the programs claimed to use explicit instruction, there was often highly variant and insufficient attention to some components, including independent practice, feedback, and monitoring.

Recent reports by national organizations corroborate this picture (e.g., EdReports, What Works Clearinghouse [WWC]). For example, the EdReports organization assembles educator-led teams to conduct reviews of curriculum using standards they have developed. The findings from these reviews are published on the website; there is variability in the findings, with some curriculum being aligned with the organization’s standards and other curriculum lacking evidence of alignment (EdReports Explore Reports, 2024). Further, mainstream media reports (e.g., Gewertz, 2020; Goldstein, 2020; Hanford, 2018, 2019; Wexler 2020) have recently brought national attention to this lack of alignment between commonly used core literacy curricula and literacy research. Together, these findings and reports have prompted states to enact literacy legislation related to curriculum review and adoption as tools for reforming educational practice and improving student outcomes (Schwartz, 2022).

The quality of curricula plays a crucial role in student outcomes and therefore a state’s or district’s choice of a curriculum stands to impact student learning (Agodini et al., 2009; Preschool Curriculum Evaluation Research Consortium (PCERC), 2008; Whitehurst, 2009). Curricula evaluations or reviews can be useful for divisions who adopt programs, teachers who implement them, and other interested parties, such as families. However, reviewing curricula is not straightforward, and no one “gold standard” approach exists. One challenge is that research has yet to definitively identify the factors that make a curriculum effective (Steiner et al., 2019). Without this understanding, it can be difficult to determine appropriate evaluation criteria. Additionally, evaluative processes vary significantly, and research is still in the early stages of assessing the reliability and validity of the different curriculum review tools available (e.g., Reading League Curriculum-Evaluation Guidelines; Burns et al., 2024).

## Aspects of curriculum review materials

This study focuses on (a) literacy content domains, (b) content-agnostic instructional practices (i.e., pedagogy), and (c) contextual information addressed in core literacy curriculum review materials (i.e., evaluation systems, such as rubrics and checklists, as well as accompanying process documents) for kindergarten through second grade (K-2). These aspects are described in the following sections.

### Content

Literacy, an evolving lifelong process, is defined as the ability to read, write, and communicate effectively, allowing individuals to fully engage in society (National Coalition for Literacy, n.d.). The interactive dynamic literacy model suggests that reading and writing share underlying skills and knowledge (Kim, 2020). One of the most universally utilized frameworks for understanding reading development is the simple view of reading (Gough & Tunmer, 1986). This framework has been empirically validated with both monolingual English-speaking and multilingual English-learner students across elementary and secondary grades (Florit & Cain, 2011; Lonigan et al., 2000). The simple view of reading posits that reading comprehension is not possible without the simultaneous presence and interaction of the two constructs: word reading and linguistic comprehension. Both constructs rely on a set of subskills. Word reading or code-related subskills include phonological awareness (PA), phonics/decoding, encoding, and high-frequency words. Linguistic comprehension or meaning-related subskills include background/world knowledge, vocabulary, and oral language. Other models, including the direct and inferential mediation model of reading comprehension (DIME; Cromley et al., 2010) and the direct and indirect effects model of reading (DIER; Kim, 2017), also emphasize the importance of these subskills for reading fluency and reading comprehension. Additionally, WWC practice guides on *Foundational Skills to Support Reading for Understanding in Kindergarten Through 3rd Grade* (Foorman et al., 2016) and *Improving Reading Comprehension in Kindergarten Through 3rd Grade* (Shanahan et al., 2010) recommend that all these subskills be included within core literacy instruction. The Shanahan et al. (2010) practice guide, focused on reading comprehension, also emphasizes the importance of reading comprehension strategies instruction and the use of text structure to comprehend, learn, and remember content. Lastly, the WWC practice guide on *Teaching Elementary School Students to Be Effective Writers* (Graham et al., 2012) emphasizes the importance of writing, including handwriting, spelling, and sentence construction, within core literacy instruction. As such, this study focuses on the literacy domains of PA, phonics/decoding, encoding, high-frequency words, background/world knowledge, vocabulary, oral language, fluency, reading comprehension, comprehension strategies, text structure, and writing.

### Pedagogy

Practice guides and national and state standards clearly delineate the literacy skills and knowledge that students should acquire (e.g., Foorman et al., 2016; Shanahan et al., 2010). However, there is less consensus on how to teach students (i.e., the instructional methods that should be used) within these domains. Although identifying criteria for identifying evidence-based pedagogical approaches can be challenging, research has identified some content-agnostic literacy instructional practices that are more effective than others. For example, literature reviews, syntheses, and meta-analyses (e.g., Archer & Hughes, 2011; Ehri et al., 2001; Hughes et al., 2017) consistently underscore the benefits of explicit instruction (i.e., instruction that follows a gradual release of responsibility model, moving from teacher-led modeling with clear explanations, to guided practice, and eventually leading to student independence) across literacy content domains (e.g., word knowledge and comprehension). Similarly, an abundance of evidence suggests that students benefit from systematic instruction (i.e., the presence of a logical sequence of instruction that facilitates students’ learning by layering previously learned concepts onto existing knowledge and reinforcing connections between content; Ehri et al., 2001; Reigeluth & Merill, 1979).

Additionally, students often benefit from multiple opportunities to practice a new skill and receive teacher scaffolding in order to achieve proficiency (Archer & Hughes, 2011; Dunlosky et al., 2013). Students learning English as a second language also benefit from instruction that is specially designed to support the development of their listening, speaking, reading, and writing skills in English (Baker et al., 2014; Goldenberg, 2008). Teachers can support all students by structuring learning activities and tasks to offer diverse distributed, cumulative, and interleaved practice opportunities, including opportunities for review (Hughes & Lee, 2019). Many of these practice opportunities involve the use of student-facing materials (e.g., various types of texts, manipulatives, workbook pages). These materials are most effective when they are engaging and relevant to the students. Further, diverse student representation within the curriculum materials, specifically student-facing materials, is important for fostering inclusivity, promoting cultural responsiveness, and ensuring all students can see themselves reflected in learning experiences and therefore connected to the learned content (Ladson-Billings, 1995).

Lastly, assessment practices within a core curriculum serve as a critical component to monitor student progress and inform instructional decisions (Fuchs & Fuchs, 2006). By providing data that reveals areas in which students may need additional support or opportunities for extension, assessment enables teachers to make instructional adjustments. Regular assessments can provide insight into how effectively a core curriculum is achieving the intended goal and contributing to student growth. As such, this study focuses on the instructional practices of explicit instruction in word knowledge, explicit instruction in comprehension, incorporation of a scope and sequence, provision of scaffolding, provision of supports for multilingual learners, provision of review opportunities, incorporation of student-facing materials, incorporation of materials that represent diverse individuals and perspectives, and incorporation of assessments.

### Contextual information

The evaluation systems (e.g., rubrics, checklists) used in the curriculum review process may be accompanied by process documents. These process documents may, for example, provide a history of state literacy legislation, outline the research underpinning evaluation indicators, or offer guidance on forming review teams and conducting the review. Together, these review materials (i.e., evaluation systems and process documents) may seek to ensure not only that particular content foci and pedagogy are addressed in core literacy curricula, but also that the curricula themselves reflect research evidence supporting curriculum components. Additionally, given that curriculum review materials are specific to each state, they may also seek to ensure alignment with state standards. Finally, some states may require that curricula be evaluated and shown to be evidence-based by independent review organizations, such as EdReports and the What Works Clearinghouse ESSA Tiers of Evidence. Although these things can ensure that curricula schools adopt are rigorous and relevant to the needs of educators and students, not much is known about the degree to which states’ curriculum review materials address them. As such, this study focused on reference to research evidence supporting the curriculum, reference to state standards, and reference to reviews or standards published by a national curriculum review organization.

### Study purpose

Core literacy curricula used in K-2 classrooms reflect widely differing degrees of alignment with research evidence (Seidenberg et al., 2020). This is problematic because high-quality literacy curricula play an important role in improving students’ literacy outcomes (Snow et al., 1998). Within the last two decades, many states have enacted literacy-related legislation with the goal of improving national literacy outcomes for students (Neuman et al., 2023). In alignment with this legislation, some states have undertaken a curriculum review process. State-level curriculum review processes have the potential to increase the degree to which children in the U.S. receive evidence-based instruction.

The aim of this study was to determine which states engage in review of K-2 core literacy curricula and to examine the materials they use to identify high-quality curricula. To gain a better understanding of cross-state differences in review materials, the research team conducted a content analysis (Krippendorff, 2013) of available curriculum review materials. We answered the following research questions:

1. Which states have a publicly-available approved K-2 core literacy curriculum list and publicly-available K-2 core literacy curriculum review materials (i.e., evaluation systems, such as rubrics and checklists, as well as process documents)?
2. What literacy content domains (e.g., PA, phonics/decoding, vocabulary) are addressed in curriculum review materials?
3. What content-agnostic instructional practices (e.g., explicit instruction, review opportunities) are included in curriculum review materials?
4. Do curriculum review materials reference (a) research evidence supporting the curriculum, (b) state standards, or (c) reviews or standards published by a national curriculum review organization (e.g., EdReports, What Works Clearinghouse ESSA Tiers of Evidence)?

## Method

The research team first systemically identified K-2 core literacy curriculum review materials that matched our inclusion criteria. We then conducted a content analysis to critically analyze curriculum review materials. We sought to identify characteristics of materials in a systematic way that could be reproduced by others (Krippendorff, 2013).

### Data sources

The documents identified and analyzed in this study were state K-2 core literacy curricula review materials (i.e., evaluation systems, such as rubrics and checklists, as well as accompanying process documents) publicly available on U.S. states’ department of education websites in October 2024. Specifically, documents were included in this content analysis if (a) a state-approved core literacy curriculum list for K-2 students and (b) core literacy curriculum review materials were made publicly available on the state department of education website*.*

To gather the most accurate and current documents, the first and second authors independently conducted searches of state department of education websites for all 50 states. The authors each used the same two sets of search terms in Google, one for identifying published approved lists and one for identifying curriculum review materials: (a) *[STATE] department of education reading approved curriculum list or high-quality instructional materials approved list* and (b) *[STATE] department of education reading and/or literacy curriculum review materials rubrics and/or checklists*. There were two disagreements in the documents identified for review by the researchers. In both instances, different documents were located by each researcher during the independent search process; the researchers had to determine which was the most current document to reference. After discussion, consensus was reached about the correct document to include in the analysis. The above search method resulted in documents from 23 states being identified for inclusion in the content analysis.

Among states with documents that were not initially identified for inclusion were four states (Alaska, Arizona, California, and North Dakota) that had published lists of approved curricula, but the research team was unable to locate the review materials used to generate the lists. The first author sent email requests to each of the four states’ departments of education inquiring about the availability of their curriculum review materials. One state (California) responded with directions for accessing their publicly available materials; the other three states did not respond. Given that the purpose of the study was to learn about the curriculum review materials of states with transparent processes, we were not able to analyze materials for those three states. As a result, curriculum review materials for 24 states were included in this study.

Individual OneDrive folders were created for each of the 24 included states. The first author uploaded PDFs of any curriculum review materials and accompanying process documents to the individual folders to prepare for the analysis. In one case, the materials could not be downloaded as a PDF, so the first author created step-by-step instructions with screenshots to guide others as to which parts of the website to focus on when coding. Coders were instructed not to access any documents outside the folders nor to click on any external links when coding.

### Coding

Coders used a multi-step, iterative process to examine the curriculum review materials and objectively describe their content (Krippendorff, 2013). First, a coding guide was created to identify and define the information to code. Codes and definitions were informed by WWC practice guides outlining evidence-based recommendations to support the development of foundational reading skills (e.g., PA, phonics/decoding), reading comprehension, and writing (i.e., Foorman et al., 2016; Graham et al., 2012; Shanahan et al., 2010) and by the Reading League’s Curriculum Review Workbook glossary (The Reading League Compass, n.d.). A total of 24 variables were coded. These codes fell into three broad categories: literacy content domains (e.g., phonics/decoding, vocabulary, reading comprehension, writing; 12 items), content-agnostic instructional practices (e.g., use of explicit instruction, provision of review opportunities; 9 items), and contextual information (e.g., alignment to state standards; 3 items). All variables were coded using a “1” for present or “0” for not present. The complete coding guide is provided in Supplemental Appendix A.

Prior to coding, all coders received training until they were able to independently code review materials with 90% inter-rater agreement with the first author. Training included explanation of the coding guide and practice coding of review materials. After the practice coding, researchers then met to discuss disagreements and refine the coding guide as needed. Defining and operationalizing overlapping concepts (e.g., vocabulary and oral language) presented the greatest challenge. Discussion about these codes led to more detailed descriptions in the coding guide. After the initial practice and refinement, researchers independently coded another state’s review materials to establish inter-rater agreement.

Once initial agreement was established, researchers continued to independently code the remaining documents. All documents were double coded. Stata (StataCorp, 2025) was used to calculate inter-rater agreement between the two coders. The average inter-rater agreement was 94%. There was 95% agreement in the literacy content domains category, 92% in the content-agnostic instructional practices category, and 94% agreement in the contextual information category. All discrepancies were resolved via discussion and consensus, and final coding reflects full agreement.

### Analysis

The researchers then used content analysis (Krippendorff, 2013) to interpret the frequency and patterns within the codes. Units of information were counted as “1” for present or “0” for not present. The remaining context and quality of the information were not evaluated in this study. The distribution of frequency across constructs (i.e., literacy content domains, instructional practices) was analyzed in comparison to evidence-based practices referred to in the IES Practice Guides. Any deviations from patterns were noticed and examined. Where misalignments were observed, these were identified as areas for potential improvement.

## Results

As shown in Table 1, we identified 24 states that have a publicly available approved K-2 core literacy curriculum list and publicly available K-2 core literacy curriculum review materials. Of the 24 states, 18 states (75%) provided a process document as part of their curriculum review materials.

**Table 1:** State information

State	Publicly available approved K-2 core literacy curriculum list	Publicly available K-2 core literacy curriculum review materials
Alabama	Y	Y
Alaska	Y	N
Arizona	Y	N
Arkansas	Y	Y*
California	Y	Y*
Colorado	Y	Y*
Connecticut	Y	Y*
Delaware	Y	Y
Florida	Y	Y*
Georgia	Y	Y*
Hawaii	N	N/A
Idaho	Y	Y*
Illinois	N	N/A
Indiana	Y	Y*
Iowa	N	N/A
Kansas	N	N/A
Kentucky	N	N/A
Louisiana	Y	Y*
Maine	N	N/A
Maryland	N	N/A
Massachusetts	N	N/A
Michigan	N	N/A
Minnesota	Y	Y*
Mississippi	Y	Y*
Missouri	Y	Y*
Montana	N	N/A
Nebraska	N	N/A
Nevada	N	N/A
New Hampshire	N	N/A
New Jersey	N	N/A
New Mexico	Y	Y*
New York	N	N/A
North Carolina	N	N/A
North Dakota	Y	N
Ohio	Y	Y*
Oklahoma	Y	Y
Oregon	Y	Y
Pennsylvania	N	N/A
Rhode Island	Y	Y*
South Carolina	N	N/A
South Dakota	N	N/A
Tennessee	Y	Y
Texas	Y	Y*
Utah	Y	Y*
Vermont	N	N/A
Virginia	Y	Y*
Washington	N	N/A
West Virginia	N	N/A
Wisconsin	Y	Y
Wyoming	N	N/A

Note: *Y* yes, *N* no, *N/A* not applicable

^*^Review materials include a process document

### Literacy content domains

We coded for the presence of 12 literacy content domains: PA, phonics/decoding, encoding, high-frequency words, background/world knowledge, vocabulary, oral language, fluency, reading comprehension, comprehension strategies, text structure, and writing. On average, states’ curriculum review materials addressed 11 of the 12 literacy content domains (range: 6–12). Five literacy content domains were addressed in all 24 states’ curriculum review materials: PA, phonics/decoding, vocabulary, fluency, and reading comprehension. Most of the 24 states’ curriculum review materials addressed the domains of oral language (23 states [96%]), background/world knowledge (22 states [92%]), writing (22 states [92%]), and encoding (21 states [88%]). Nineteen states (79%) addressed high-frequency word instruction, and 17 states (71%) addressed reading comprehension strategy instruction. Text structure was specifically addressed in 14 states’ curriculum review materials (58%).

## Content-agnostic instructional practices

We coded for the presence of nine content-agnostic instructional practices: explicit instruction in word knowledge, explicit instruction in comprehension, incorporation of a scope and sequence, provision of scaffolding, provision of supports for multilingual learners, provision of review opportunities, incorporation of student-facing materials, incorporation of materials that represent diverse individuals and perspectives, and incorporation of assessments. On average, states addressed eight of the nine content-agnostic instructional practices (range: 3–9). The use of assessments within a curriculum was the only practice present in all 24 states’ curriculum review materials. Almost all states (23 states; 96%) included metrics in their curriculum review materials that evaluated whether curricula used explicit instruction in word knowledge, provided a scope and sequence, and incorporated scaffolding practices. Similarly, 22 states (92%) included information about student-facing materials, and 21 states (88%) addressed instructional supports for multilingual learners. Explicit instruction in comprehension and opportunities for review were each addressed in 19 states’ curriculum review materials (79%). Fifteen states’ curriculum review materials (63%) included information about the use of materials that represent diverse individuals and perspectives.

### Contextual information

We coded curriculum review materials for three pieces of contextual information: reference to research evidence supporting the curriculum, reference to state standards, and reference to reviews or standards published by a national curriculum review organization. Almost all of the 24 states (22 states; 92%) referenced state standards in their curriculum review materials. Similarly, 21 states (88%) referenced research evidence in support of the curriculum. Only eight states (33%) referenced a national curriculum review organization in their curriculum review materials.

## Discussion

This study sought to learn more about the content of state-level curriculum review materials. Specifically, researchers analyzed K-2 core literacy curriculum review materials from states that published an approved list of core curricula and published the curriculum review materials that led to the list. Importantly, our analysis was not intended to determine the quality of curriculum review materials or suggest that there is one “best” way to review core curricula at the state level. Rather, the goal was to glean insights into the aspects of K-2 core literacy curriculum that states deem as important.

We determined that almost half of the states in the U.S. had curriculum review materials available. It is important to note that, anecdotally, these curriculum review materials widely varied across states. For example, their evaluation systems ranged from simple checklists, with each indicator being scored present or not (e.g., Indiana, Louisiana, Minnesota) to more complex rating scales documenting the degree to which an indicator was present (e.g., Alabama, Idaho). Some states’ materials included more extensive point systems in which indicators were weighted and could receive varying amounts of points (e.g., Mississippi, Oklahoma). Some states’ materials included a combination of evaluation systems (e.g., Missouri, Wisconsin). Additionally, some states conducted a multi-phase review process (i.e., a curriculum must pass the first phase before moving to the next) and thus had separate review materials for each phase (e.g., Arkansas, Colorado, Virginia). Moreover, the level of detail in individual indicators within an evaluation system varied across states. Most states’ indicators included short, simple statements (e.g., Idaho, Ohio), whereas other states’ indicators were more detailed, including examples or additional guidance (e.g., Oregon, Texas). Further, when present, process documents varied widely in content and detail.

Across states’ K-2 core literacy curriculum review materials, our analysis revealed that there was preliminary support of evidenced-based content and pedagogy reflected in the materials. For example, many states’ materials addressed literacy content domains that prior research have identified as being vital for literacy development, such as PA, phonics/decoding, encoding, vocabulary, oral language, fluency, reading comprehension, and writing. Instruction specifically related to high-frequency words (i.e., regular or irregular words that commonly occur in texts and are expected to be read automatically, or by sight) was less commonly addressed. High-frequency words lie along a continuum of irregularity (i.e., regularly spelled, temporarily irregularly spelled, and permanently irregularly spelled). Research has demonstrated value in drawing students’ attention to letter-sound relations to learn to read words, including regularly spelled and temporarily irregularly spelled high-frequency words (Ehri, 2020; Miles et al., 2018). As such, it may be prudent for states to include indicators focused on evidence-based high-frequency word instruction within their curriculum review materials.

Further, findings suggest that reading comprehension remains a complex instructional domain. Although overall reading comprehension is frequently addressed within the curriculum review materials, the focus of reading comprehension instruction (i.e., comprehension strategies, text structure, and background/world knowledge) remains varied. Given that there is strong evidence for teaching students how to use reading comprehension strategies and moderate evidence for teaching students to identify and use the text’s organizational structure as a means for improving comprehension (Shanahan et al., 2010; Zhang & Wijekumar, 2024), states may want to consider providing guidance around specific approaches to reading comprehension instruction.

Our analysis also revealed that many states’ K-2 core literacy curriculum review materials addressed evidence-based instructional practices, such as the use of explicit instruction (in both word knowledge and comprehension), incorporation of a scope and sequence, provision of scaffolding, opportunities for review, and supports for multilingual learners, use of assessments within the curriculum, as well as inclusion of student-facing materials. However, representation of diversity in student-facing materials was often overlooked, despite its proven importance in fostering engagement, validating cultural identities, and promoting inclusivity. Diverse texts play a crucial role in helping students see themselves reflected in the materials they encounter, which supports both academic and social-emotional development (Bishop, 1990; Brenna, 2008). As such, states might benefit from addressing representation in text in their curriculum review materials.

It is not surprising that most states’ K-2 core literacy curriculum review materials addressed research evidence in support of the curriculum nor that they involved alignment with state standards. By including explicit indicators of evidence-based content and instructional practices and alignment with state standards in their publicly available curriculum review materials, states are promoting a transparent process. This transparency allows states to set clear expectations and influence curriculum developers and publishers to produce materials that reflect these expectations. When publishers are aware of consistent, non-negotiable expectations—such as explicit instruction across all domains, built-in formative assessments, and equity-focused representation in student-facing materials—they are more likely to embed these elements into their curricula to remain competitive in the marketplace. Thus, the curriculum review criteria that states implement may influence curriculum development and the success of curriculum companies.

Some states incorporated evaluations from national curriculum review organizations, such as EdReports and What Works Clearinghouse ESSA Tiers of Evidence, into their state-level curriculum review processes. In some cases, states (e.g., Arkansas, Ohio, Rhode Island) required curricula to achieve a specific rating from these organizations before advancing to evaluation with a state-specific review material. Other states (e.g., Connecticut, Florida) integrated findings from these national reviews as a component of their curriculum review process. It is possible that states that use information from national review organizations may see greater consistency between state-level evaluation outcomes and the ratings provided by these organizations. Conversely, some states may opt not to utilize these external reviews in part due to differences in evaluation criteria and review procedures, which may not align with state-specific priorities or requirements.

Overall, there were some common themes that emerged across states’ K-2 core literacy curriculum review materials, including the presence of evidenced-based content (e.g., PA, phonics/decoding, vocabulary, fluency, and reading comprehension) and pedagogy (e.g., explicit, systematic, and assessment-informed instruction). There were also some notable differences, such as variability in literacy content domains addressed (e.g., instruction in comprehension strategies and text structure) and variability in instructional practices addressed (e.g., explicit instruction in comprehension and opportunities for review).

One implication of this variability across curriculum review materials is that the approved list of K-2 core literacy curricula that states produce may also vary. Variability in state-approved curriculum lists could raise concerns about the consistency and reliability of the curriculum review process, including curriculum review materials. Although this content analysis sheds light on the presence of specific indicators within curriculum review materials, it does not address their frequency, emphasis, or conceptualization. The weighting of indicators, such as encoding instruction being minimally represented in one state but heavily emphasized in another, can significantly impact how curricula are assessed and prioritized. For instance, a curriculum highly rated in one state may receive a lower evaluation elsewhere, complicating the understanding of what constitutes a high-quality core literacy curriculum.

As previously stated, curricula review is one small piece of a larger process comprised of multiple elements and influenced by various interested parties. Once a state required or recommended list of curricula is established, school divisions proceed with the adoption process, and teachers take on the critical role of implementing the program. Although abundant research exists on early reading and writing learning and instructional practices, the gap between research and practice persists (Solari et al., 2020). Teachers require opportunities to understand the research and its effectiveness, and how to practically apply it in their classrooms. Successful implementation involves not only fidelity to the chosen instructional materials but also the discernment to adapt or exclude certain aspects when necessary to impact student outcomes. Importantly, high-quality curricula alone are insufficient without intentional and well-supported implementation strategies. By prioritizing professional development and ongoing support during the implementation phase, curricula can potentially serve as a powerful tool to bridge the research-to-practice gap.

## Limitations and directions for future research

Curricula review materials represent one aspect of the larger process of review and implementation. This study aimed to observe and document the current practices, rather than to comprehensively analyze or evaluate entire processes. The current study’s findings are limited in relation to the methods used to identify and analyze K-2 core literacy curriculum review materials. Due to the ever-changing landscape of state policy, the findings from this study are time-limited because they reflect the content of the review materials available at the time of our analysis. States may revise their review materials on an ongoing basis (e.g., to reflect current research) or additional states may undergo a curriculum review process (e.g., as new legislation is enacted). Openness in the curricula review process may also evolve over time. Anecdotal evidence suggests that some states offer easy-to-navigate websites with materials readily accessible to the public, whereas others have more cumbersome systems that require more navigation to locate materials. These systems may be updated over time, resulting in easier access to more materials.

Furthermore, this study focused on materials for reviewing curriculum for students in kindergarten through second grade. However, several states are also reviewing materials for students in upper-elementary grades as well as middle school. Additional research examining the review materials in these grades and an analysis of the shift of focus between grades could contribute to a more complete picture of the curricula review processes.

Lastly, state-level curriculum review materials are just one component of a broader, under-researched system. The curriculum review process itself warrants greater examination as critical questions about who participates on review teams, how reviews are conducted, and the reliability of evaluation systems remain. Not all states offer the same level of transparency in the process and therefore pose a challenge in analyzing components. In a recent study of The Reading League Curriculum-Evaluation Guidelines (The Reading League Compass, n.d.), Burns et al. (2024) highlighted the lack of research validating metrics used to review curricula. Their findings underscored the need for greater rigor in developing and evaluating curriculum review tools.

## Conclusion

The recent wave of literacy-focused legislation and core curriculum review efforts reflects a nationwide commitment to improving literacy outcomes and addressing opportunity gaps among K-2 students. By aligning instructional materials with research-based practices and incorporating comprehensive review processes, states aim to provide educators with curricula material that is both effective and equitable. Although these efforts signify progress towards improving outcomes for all students, further examination of curriculum review materials and the processes in which they are used is crucial to ensure clarity and consistency for educators, policymakers, and publishers. The findings from this study show that although many evidence-based content and practices are present, there remains variability across states with some domains more represented than others. With sustained focus on evidence-based approaches and cross-state collaboration and alignment, there exists the potential to create meaningful and lasting improvements in literacy instruction and outcomes for all students.

## Supplementary Information

Below is the link to the electronic supplementary material.

Supplementary file1 (DOCX 16.4 KB)
