# Supplementary material for: Content analysis of state-level review materials for K-2 core literacy curricula
Source: Ann Dyslexia. 2025 Jun 5;75(3):547–63. doi: 10.1007/s11881-025-00334-1 (PMC12662850; doi:10.1007/s11881-025-00334-1)
Supplement: Supplementary file 1 — Supplementary file1 (DOCX 16.4 KB) [file 11881_2025_334_MOESM1_ESM.docx]

**Supplemental Appendix A**

*Coding Guide*

**State Review Material Content Analysis**

**Coding Procedures Guide 2024**

Unless specified otherwise, only enter numbers in each cell. In most cases, 0 = No and 1 = Yes. Do not leave any blanks.

**Procedures in Order of Appearance on Code Sheet**

**State:** Record the state that developed the document(s) you are viewing.

**Process Document:** Indicate yes (1) if there is a process document that accompanies the curriculum review materials and no (0) if there are no process documents present. A process document is any sort of documentation that outlines how the review should be conducted, provides additional information to accompany the scoring system, or further explains terminology used in the review materials. These documents will be housed in the folder. Do not access additional documents or information outside of the folder.

**National Curricula Review Organization:** Indicate yes (1) if the state review process (as outlined in the process documents or scoring materials) includes seeking input or referencing scores from outside national curriculum review organizations, including but not limited to ESSA, EdReports, and no (0) if not.

**Research Base:** Indicators that require evidence of a research base for the curriculum. This may include indicators that want vendors to cite studies that show positive effects of the program itself, or studies that show a particular component (e.g., systematic phonics instruction) is beneficial. Indicate yes (1) or no (0). Broad descriptors such as “research-aligned practices” or “aligns with current research” or “reflects best practices” should be coded as a (1).

**State Standard Alignment:** Indicators that check for alignment between curriculum and state standards. Indicate yes (1) or no (0).

**Instructional Practices. These refer to "content agnostic” instructional practices. Indicate yes (1 = yes) if the curriculum review materials include an item or indicator about the practice and no (0 = no) if not.**

**Explicit Instruction of word knowledge skills (i.e., encoding, decoding, high frequency words, fluency, and p.a.):** Indicators that focus on the development of word knowledge using instruction that follows a gradual release of responsibility model, moving from teacher-led modeling with clear explanations, to guided practice, and eventually leading to student independence. Indicator **must** include the words “explicit instruction” or “gradual release” or “I do. We do. You do.” or “structured teaching” or “direct instruction.”

**Explicit Instruction of language and/or reading comprehension skills (i.e., vocabulary, reading comprehension strategies, text structure, background/world knowledge, and language.):** Indicators that focus on the development of language and/or reading comprehension using instruction that follows a gradual release of responsibility model, moving from teacher-led modeling with clear explanations, to guided practice, and eventually leading to student independence. Indicator **must** include the words “explicit instruction” or “gradual release” or “I do. We do. You do.” or “structured teaching” or “direct instruction.”

**Scope and Sequence of word knowledge skills (i.e., phonics and P.A.):** Indicators that look for an outline that describes a progression of skills to be taught over the course of a unit or a year, using a l*ogical sequence* that builds from simple to complex. May also be referred to as “sequential instruction.”

**Scaffolds:** Indicators that address task adjustment (e.g., what to do if a student is not understanding a task) and/or corrective teacher feedback.

**Supports for Multilingual Learners:** Instruction that is specially designed for multilingual learners to develop their listening, speaking, reading, and writing skills in English.

**Opportunities for Review:** Indicators look for elements of review. Evidence may be found in the scope and sequence, warm-up tasks, etc.

**Student-Facing Materials:** Indicators that address appropriateness, quality, or quantity of any student-facing material, including decodable texts, books read aloud, grade-level passages, grade-level books, workbooks, manipulatives, etc.

**Representation in Text**: Indicators that look at student-facing materials for the inclusion of various perspectives and representation of a wide range of experiences, different demographics, cultures, and physical characteristics encompassing all aspects of human diversity.

**Assessment:** Indicators that look if the program includes or supports teachers use of formative and/or progress monitoring assessment of individual reading components (i.e., decoding, P.A., encoding, fluency, vocabulary, comprehension). The presence of assessment in any area should be marked with a 1.

**Content Indicators. These refer to specific literacy content. Indicate yes (1 = yes) if the curriculum review materials include an indicator about the content and no (0 = no) if not.**

**Phonological Awareness:** Awareness of the phonological structure of words in one’s language. A term that encompasses awareness of *individual words in spoken sentences*, *syllables*, *onsets or rimes,* and *individual phonemes.*

**Decoding:** Sounding out words using letter-sound knowledge and *blending* those sounds together to pronounce the word. Includes isolated and in-text scenarios.

**Encoding:** *Spelling* words using phonemic awareness and knowledge of letter sounds. Indicator must use the words encoding and/or spelling.

**High-Frequency Words:** Reading commonly used words that are both regular and irregular for the grade level. May also be referred to as sight words.

**Background Knowledge/World Knowledge:** Knowledge needed to comprehend a particular text. Making connections between prior knowledge/learning and new learning and/or building knowledge prior to beginning a new unit/concept/task.

**Vocabulary:** Building students’ receptive and expressive word bank through lessons and tasks that involve students seeing and utilizing the written form of the word.

**Conversation and Language:** Facilitating conversation and/or discussions (e.g., whole class, small group, or partners) or intentionally developing other aspects of oral language (e.g., syntax, pragmatics, grammar, morphology). The primary focus of the indicator is on the development and instruction of oral language skills. Opportunities for students talk, alone, do not constitute a 1.

**Fluency:** *Rate, prosody and/or accuracy* of student’s *connected text reading*. May also include *automaticity* tasks.

**Reading Comprehension:** Ability to understand, interpret, and analyze written text. This is a broad domain encompassing several underlying constructs. Use this code if reading comprehension is referred to broadly WITHOUT references specific comprehension strategies, text structure, or background knowledge/world knowledge. *Note: This code should be marked 1 if any of the following codes are marked 1: reading comprehension strategies, text structure, background knowledge/world knowledge.*

**Reading Comprehension Strategies:** Strategies that focus on making meaning from text (e.g., inferencing, summarizing, visualizing).

**Text Structure:** Using text structure as a comprehension support (e.g., cause/effect, problem/solution, description, sequence/chronological order, compare/contrast).

**Writing:** Composition, handwriting, letter formation, and writing in response to reading.
